# Strategies and Lessons Learned During Cleaning of Data From Research Panel Participants: Cross-sectional Web-Based Health Behavior Survey Study

**DOI:** 10.2196/35797

**Published:** 2022-06-23

**Authors:** Mariana Arevalo, Naomi C Brownstein, Junmin Whiting, Cathy D Meade, Clement K Gwede, Susan T Vadaparampil, Kristin J Tillery, Jessica Y Islam, Anna R Giuliano, Shannon M Christy

**Affiliations:** 1 Department of Health Outcomes and Behavior Moffitt Cancer Center Tampa, FL United States; 2 Department of Biostatistics and Bioinformatics Moffitt Cancer Center Tampa, FL United States; 3 Department of Oncological Sciences University of South Florida Tampa, FL United States; 4 Department of Public Health Sciences Medical University of South Carolina Charleston, SC United States; 5 Department of Genitourinary Oncology Moffitt Cancer Center Tampa, FL United States; 6 Department of Gastrointestinal Oncology Moffitt Cancer Center Tampa, FL United States; 7 Center for Immunization and Infection Research in Cancer Moffitt Cancer Center Tampa, FL United States; 8 Participant Research, Interventions, and Measurement Core Moffitt Cancer Center Tampa, FL United States; 9 Department of Cancer Epidemiology Moffitt Cancer Center Tampa, FL United States

**Keywords:** data cleaning, data management, data integrity, quality assessment, research panel, web-based survey, interdisciplinary research, surveys and questionnaires, health behavior, internet

## Abstract

**Background:**

The use of web-based methods to collect population-based health behavior data has burgeoned over the past two decades. Researchers have used web-based platforms and research panels to study a myriad of topics. Data cleaning prior to statistical analysis of web-based survey data is an important step for data integrity. However, the data cleaning processes used by research teams are often not reported.

**Objective:**

The objectives of this manuscript are to describe the use of a systematic approach to clean the data collected via a web-based platform from panelists and to share lessons learned with other research teams to promote high-quality data cleaning process improvements.

**Methods:**

Data for this web-based survey study were collected from a research panel that is available for scientific and marketing research. Participants (N=4000) were panelists recruited either directly or through verified partners of the research panel, were aged 18 to 45 years, were living in the United States, had proficiency in the English language, and had access to the internet. Eligible participants completed a health behavior survey via Qualtrics. Informed by recommendations from the literature, our interdisciplinary research team developed and implemented a systematic and sequential plan to inform data cleaning processes. This included the following: (1) reviewing survey completion speed, (2) identifying consecutive responses, (3) identifying cases with contradictory responses, and (4) assessing the quality of open-ended responses. Implementation of these strategies is described in detail, and the Checklist for E-Survey Data Integrity is offered as a tool for other investigators.

**Results:**

Data cleaning procedures resulted in the removal of 1278 out of 4000 (31.95%) response records, which failed one or more data quality checks. First, approximately one-sixth of records (n=648, 16.20%) were removed because respondents completed the survey unrealistically quickly (ie, <10 minutes). Next, 7.30% (n=292) of records were removed because they contained evidence of consecutive responses. A total of 4.68% (n=187) of records were subsequently removed due to instances of conflicting responses. Finally, a total of 3.78% (n=151) of records were removed due to poor-quality open-ended responses. Thus, after these data cleaning steps, the final sample contained 2722 responses, representing 68.05% of the original sample.

**Conclusions:**

Examining data integrity and promoting transparency of data cleaning reporting is imperative for web-based survey research. Ensuring a high quality of data both prior to and following data collection is important. Our systematic approach helped eliminate records flagged as being of questionable quality. Data cleaning and management procedures should be reported more frequently, and systematic approaches should be adopted as standards of good practice in this type of research.

## Introduction

The use of web-based methods to collect population-based data has burgeoned over the past two decades [1,2]. In fact, the number of published manuscripts reporting use of data from web-based platforms and research panels increased from 1 in 2010 to over 1200 in 2015 [3,4]. Research panels consist of individuals who volunteer to be contacted about potential participation in research studies [5,6]. Often, these research studies are available to potential participants via web-based platforms and may offer incentives for participation [5,6]. Researchers have used web-based platforms and research panels to study a wide variety of topics, including smoking cessation [7-9], social and behavioral determinants of health [10], eating habits [11], treatment seeking behaviors [12], social media use and experiences [13], participation in clinical trials research [14], virtual harassment and cyberbullying [15], addiction research [16,17], and infectious disease prevention behaviors [18,19], among others.

Web-based platforms and research panels are useful tools for recruiting and collecting information from large participant samples in a relatively short amount of time. More recently, these have become an alternative method for data collection due to COVID-19 pandemic restrictions (eg, social distancing). For example, after the start of the COVID-19 pandemic, one company with a research panel that allows researchers to reach potential participants via a web-based platform reported a 400% increase in the number of researchers using their platform [20]. Advantages to using these web-based platforms and research panels include the ability to assess a variety of behaviors, a high degree of diversity among potential participants, potentially lower research coordination costs, decreased time in data collection, and the ability to reach populations that otherwise would be difficult to recruit [6]. The number of users in these platforms and research panels have also increased, in part, due to the availability and ease of participation in research, the need to find supplementary income, or simply, for monetary gain [20]. However, fraudulent responses resulting from careless answering and the use of virtual personal networks to mask identities have contributed to a decline in data quality and integrity [21-23].

Only a few researchers have published their recommendations to improve the integrity of web-based survey data, and a combination of different strategies is advised [24-26]. Data integrity can be defined as the expectation of quality that is satisfactory and suitable to answer a research question [27]. In 2004, the Checklist for Reporting Results of Internet E-Surveys (CHERRIES) was published as a recommendation to ensure adequate reporting of web-based surveys [28]. One of the CHERRIES guidelines encourages researchers to report prevention methods for multiple survey entries from the same respondent, such as checking duplicate IP addresses and use of cookies. Although CHERRIES is helpful for improving researchers’ reporting of findings from web-based survey studies, other data cleaning strategies to assess data quality are not specified in the guidelines.

In this paper, our study team shares our experiences in data cleaning to improve data quality and integrity from a web-based survey that recruited participants via a research panel. Our interdisciplinary team used a systematic and detailed data cleaning approach prior to the analyses. The goal for this paper is to describe our team’s process and to share lessons learned, including a checklist developed by the team that other research teams could use or adapt to guide their data cleaning process.

## Methods

### Overview

Data for this study were collected from panelists, either directly or via verified partners, of a research panel available for scientific and marketing research. The goal of our web-based survey was to examine human papillomavirus (HPV) and HPV vaccine knowledge, beliefs, attitudes, health care experiences, vaccine uptake, vaccination intentions, and other health behavior constructs, as well as information sources, preparedness for shared decision-making, and preferences that could help inform future HPV vaccination educational interventions for age-eligible individuals. Our interdisciplinary research team was composed of individuals with academic training in biostatistics, public health, nursing, psychology, epidemiology, and behavioral oncology.

Recruitment occurred from February 25 to March 24, 2021. The target sample for the study was 4000 individuals aged 18 to 45 years, stratified with equal recruitment by the cross-tabulation of age (18-26 years vs 27-45 years) and sex at birth (male vs female). Participation was limited to individuals who were panelists, directly or through verified partners of the research panel; were aged 18 to 45 years; were living in the United States; were proficient in the English language; and had access to the internet. Our interdisciplinary team aimed to recruit a sample that was representative of the geographic as well as racial and ethnic characteristics in the United States. Florida residents and those within our cancer center’s catchment were oversampled (ie, 500 Florida residents and 3500 residents of other states) with the aim of informing future research and outreach activities. The survey was pretested by three individuals who completed the paper-based version of the survey to estimate the completion time and provide feedback on survey item wording. Based on pretesting, it was estimated that the survey would take approximately 30 minutes to complete, depending on the sequential flow of the survey (ie, skip and contingency question patterns for some individuals).

**Figure 1:**
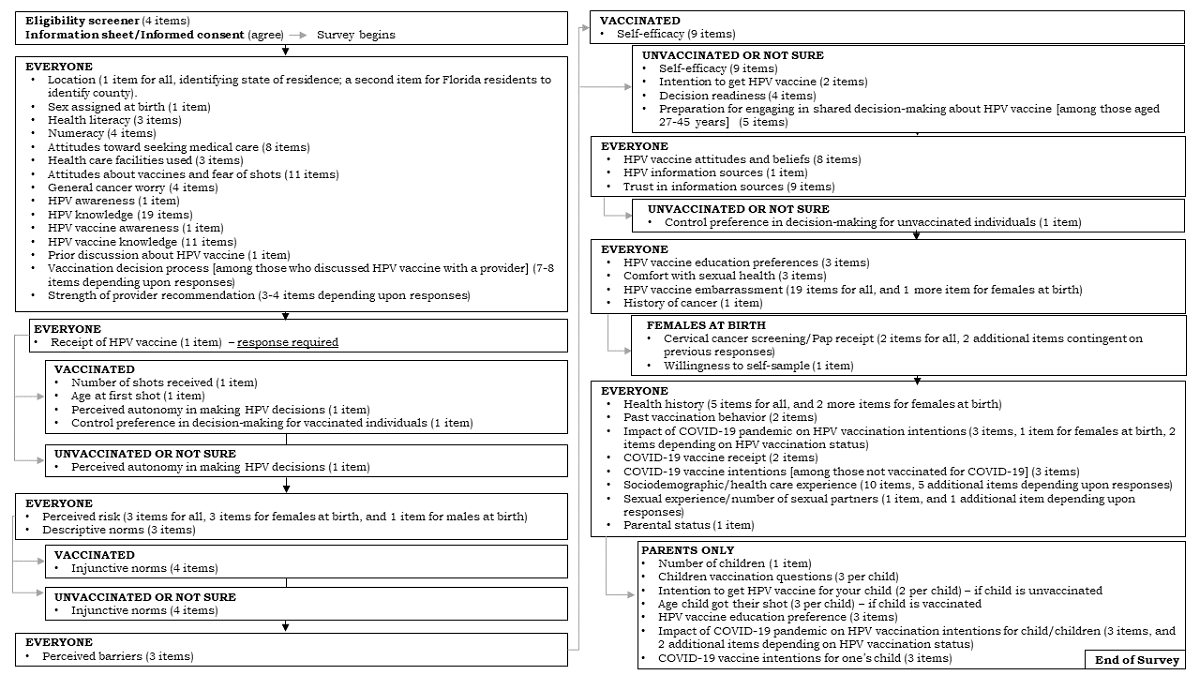
Survey schema illustrating branching logic, survey title, and number of items.

The one-time survey was programmed in Qualtrics XM [29] by a member of the study team (KJT). The survey programmer applied Qualtrics features to monitor and set quota limits for gender and age counts. The final survey contained over 200 items. The number of items displayed for each respondent depended on the survey’s branching logic, which was based on characteristics such as the respondent’s age, sex assigned at birth, HPV vaccination status, and parental status (Figure 1). For example, the programmer set the branching logic such that respondents who self-identify as parents would receive a subset of questions regarding their children’s health care experiences. Based on information that the panel company had about age, gender, and geographic location, potential participants were sent an invitation to participate in the study with a link to the survey directly by the research panel company. Individuals who were interested in participating completed a brief eligibility screener. Those who were eligible reviewed an informational sheet (ie, informed consent); eligible and interested individuals then proceeded to the main survey.

### Ethical Considerations

The Scientific Review Committee at Moffitt Cancer Center and the Institutional Review Board of record (Advara) reviewed the study and approved it as exempt (Pro00047536).

### Data Cleaning Strategies

#### Overview

Data quality is often defined in relation to aspects such as accuracy, completeness, validity, and conformity [30,31]. Evaluating the quality of web-based survey data and completing a data cleaning process before conducting statistical analyses is an important step that is often not reported transparently by research teams. To inform this process, our team conducted a literature review to identify key sources describing methods to support integrity of web-based survey responses. Findings from the literature review guided our team’s decisions and helped us reach consensus on the number and types of strategies we would employ. Then, a systematic and sequential multi-strategy plan was developed to assess responses. The plan included the following: (1) reviewing survey completion speed [24,32], (2) identifying consecutive responses [32-34], (3) identifying cases with contradictory responses [35], and (4) assessing the quality of open-ended responses [26,35]. Thus, we defined high-quality data as survey data that had been stripped of instances of consecutive identical answers, contradictory responses, nonsensical open-ended responses, and responses completed in an unrealistic amount of time (see the steps below for further details on each criterion).

#### Step 1: Duration of Survey Completion

To identify a range of survey completion durations, a group of six individuals completed the survey. As previously mentioned, prior to survey launch, three individuals who were naïve to the survey items completed paper-and-pencil versions drafts of the survey to evaluate how long it might take potential participants to complete the questions and provide feedback on item wording. Based upon the amount of time it took these individuals to complete the survey, our team anticipated that it would take participants an average of 30 minutes to complete the survey. However, we recognized that it could take some respondents less or more time to complete it depending on their responses and the corresponding skip logic that was programmed into the survey. For example, an individual who responded that they had received the HPV vaccine would receive items relevant to prior vaccine receipt, whereas an individual who reported that they had not received the HPV vaccine would receive questions about intentions to receive the HPV vaccine. Similarly, respondents with children would receive additional questions about HPV vaccination intentions for their children, whereas childless respondents would not receive those questions. Following finalization of the survey and the Qualtrics programming, an additional three team members completed the electronic (ie, Qualtrics) version of the survey.

Completion times of the Qualtrics-programmed survey within our team ranged from 5 minutes (when mindlessly and quickly clicking through the survey, but not actually reading the items) to 10 minutes (when reading and answering quickly, but legitimately) to 28 minutes (when attending to the items and reading thoroughly). Based on these test runs, consideration of the survey length and skip logic, and our best judgement, the team decided that a 10-minute (600-second) cutoff was the least amount of time that was still realistic in which respondents could take the survey while legitimately reading the items (see Table S1 in Multimedia Appendix 1 for descriptive information for Step 1).

#### Step 2: Consecutive Identical Responses

Consecutive identical responses (ie, “straight-lining” [34]) were assessed using four instruments that contained reverse-coded items and had been displayed to participants in table formats within the web-based survey. Selecting scales with reverse-coded items ensures that participant responses should not be identical in all items of a scale (see Table S1 in Multimedia Appendix 2 for a description of scales used in this step). Based on literature recommendations, we assessed consecutive identical responses of the response anchor extremes (ie, “strongly agree” and “strongly disagree”) in the instrument’s response scales [36,37]. For example, we identified records that demonstrated patterns of consecutive responses [34] on a vaccine attitudes scale, recognizing that individuals are unlikely to strongly agree or strongly disagree with both of the following statements: “Vaccines are generally safe” and “Vaccines are dangerous” [38]. A stepwise process was used to examine patterns of consecutive responses in four selected instruments: first, the instrument containing the largest number of items (ie, 19 items) was examined for patterns of consecutive responses, then survey records that failed this check were removed. These steps were repeated in the next scale, until all four scales had been checked. Records meeting those criteria were identified and removed using code in SAS software (version 9.4; SAS Institute Inc) [39] (Multimedia Appendix 3).

#### Step 3: Conflicting Responses

The team identified survey items that could indicate logical contradictions or extremely rare cases by carefully reviewing survey items and assessing patterns of responses for logical consistency. Depending on the survey content of a particular project, the types and numbers of questions used for assessment of conflicting answers might be different. For example, surveys might include the same question in two different locations of the survey (eg, age) to check for potential contradictory responses. During our data cleaning process, we decided to examine respondents who had indicated all of the following: (1) they were married or widowed or divorced, (2) they did not self-identify as asexual, (3) they reported that they had not ever had sexual intercourse (ie, vaginal, anal, or oral sex), and (4) they responded that they were a parent of one or more children. This group of cases was selected because we believe that it is an extremely unlikely scenario (ie, that one would be married or have a history of being married and have a child or children while reporting that they had never had sexual intercourse and did not self-identify as asexual) that is most likely due to careless answering. Records meeting those criteria were identified and removed using code in SAS software (version 9.4; SAS Institute Inc) [39] (Multimedia Appendix 4).

#### Step 4: Quality of Open-Ended Responses

Two team members independently assessed the quality of open-ended responses by checking all open-ended variables and identifying gibberish (ie, unintelligible responses), nonsensical responses (eg, responses that did not make sense in the context of the question asked), and patterns of identical responses within and across records (eg, exact same response to multiple open-ended items). Our team completed this in two steps. The first reviewer conducted a visual examination of cases that contained gibberish and duplicate responses. These records were flagged and removed. The second reviewer did the following: (1) identified nonsensical responses, (2) identified irrelevant responses, and (3) checked for repetitive patterns within and across records (ie, to identify whether different records had the same response patterns, because this could indicate that the same person may have completed multiple surveys). To do this, a team member (ie, first reviewer) exported survey records from SAS to a Microsoft Excel spreadsheet. Another team member (ie, second reviewer) located each open-ended variable column and sorted that column to inspect each of the responses provided, row by row. The team member also inspected the records column by column to identify patterns of open-ended responses across variables. Records that met the criteria outlined above were flagged. The same procedure was repeated for each open-ended variable until all open-ended variables in the codebook had been inspected. When the second reviewer had questions about whether or not responses were nonsensical, irrelevant, or repetitive, the research team discussed and resolved them by consensus. Survey records with instances of at least one of those three checks were flagged and subsequently removed from the data set.

## Results

Table 1 describes the systematic and sequential steps leading to the final analytic sample consisting of 2722 records. About one-sixth of 4000 records (n=648, 16.20%) were flagged and removed during Step 1 (ie, survey completion duration). Descriptive statistics (ie, mean, median, and first and third quantiles) on completion duration for both the initial and final analytic samples are included in Multimedia Appendix 1. In Step 2, 7.30% (292/4000) of the records were removed because they contained evidence of consecutive responses. The SAS code for this step is included in Multimedia Appendix 3. In Step 3, 187 out of 4000 (4.68%) records were removed because we found evidence of conflicting responses. The SAS code for this step is included in Multimedia Appendix 4. In Step 4, 151 out of 4000 (3.78%) records were removed due to evidence of poor-quality open-ended responses. This final step required the most person-time effort, as some variables took up to 25 minutes to inspect. Ultimately, based on these four steps, 31.95% (1278/4000) of the responses from the original sample were removed.

We conducted descriptive statistics to characterize our sample before and after the quality assessment procedures (Table 2). The original sample was formed with equal groups based on age (18-26 years: 50%; 27-45 years: 50%) and sex at birth (females: 50%; males: 50%). After data cleaning, the final analytic sample (N=2722) contained a slightly higher number of females (55.95%) and individuals aged 18 to 26 years (50.73%). Compared to the original sample, the final sample had comparable proportions of individuals born in the United States and across sexual orientation categories. Also, in the final sample, a slightly higher proportion of respondents reported being White (68.80% original vs 71.05% final sample), non-Hispanic (81.30% original vs 83.25% final sample), childless (53.83% original vs 58.63% final sample), and from the Midwest region of the United States (20.28% original vs 21.42% final sample). Compared to the original sample, we observed a slightly lower proportion in the final sample of respondents with a graduate degree (21.20% original vs 15.76% final sample), with an annual income of US $100,000 or more (26.73% original vs 24.17% final sample), who were married (53.95% original vs 51.54% final sample), who were employed (74.33% original vs 72.56% final sample), and without health insurance (18.23% original vs 16.79% final sample). Manuscripts have been published [40], are under review, or are in preparation describing findings from this study.

**Table 1:** Steps to ensure quality of responses leading to final analytic sample.

Data quality assessment steps	All records (N=4000)	
	Records removed, n (%)	Records left, n (%)
Original sample	0 (0)	4000 (100)
Step 1: survey duration	648 (16.20)	3352 (83.80)
Step 2: consecutive identical responses	292 (7.30)	3060 (76.50)
Step 3: contradictory responses	187 (4.68)	2873 (71.83)
Step 4: quality of open-ended responses	151 (3.78)	2722 (68.05)

**Table 2:** Descriptive characteristics of the original and final samples.

Characteristic			Original sample (N=4000), n (%)^a^		Final sample (N=2722), n (%)^a^
**Age (years)**					
	18-26	2000 (50.00)		1381 (50.73)	
	27-45	2000 (50.00)		1341 (49.27)	
**Sex assigned at birth**					
	Female	2000 (50.00)		1523 (55.95)	
	Male	2000 (50.00)		1199 (44.05)	
**Race**					
	White	2752 (68.80)		1934 (71.05)	
	Black or African American	506 (12.65)		314 (11.54)	
	Other	726 (18.15)		470 (17.27)	
	Missing	16 (0.40)		4 (0.15)	
**Ethnicity**					
	Hispanic	719 (17.98)		447 (16.42)	
	Non-Hispanic	3252 (81.30)		2266 (83.25)	
	Missing	29 (0.73)		9 (0.33)	
**Born in the United States**					
	Yes	3719 (92.98)		2529 (92.91)	
	No	263 (6.58)		189 (6.94)	
	Missing	18 (0.45)		4 (0.15)	
**Education**					
	High school or less	983 (24.58)		661 (24.28)	
	Some college or associate’s degree	1152 (28.80)		870 (31.96)	
	Bachelor’s degree	1000 (25.00)		757 (27.81)	
	Graduate school	848 (21.20)		429 (15.76)	
	Missing	17 (0.43)		5 (0.18)	
**Annual Income (US $)**					
	0-19,999	521 (13.03)		331 (12.16)	
	20,000-49,999	917 (22.93)		673 (24.72)	
	50,000-74,999	765 (19.13)		558 (20.50)	
	75,000-99,999	649 (16.23)		456 (16.75)	
	≥100,000 or more	1069 (26.73)		658 (24.17)	
	Missing	79 (1.98)		46 (1.69)	
**Relationship status**					
	Married	2158 (53.95)		1403 (51.54)	
	Other	1826 (45.65)		1317 (48.38)	
	Missing	16 (0.40)		2 (0.07)	
**Employment** **status**					
	Employed	2973 (74.33)		1975 (72.56)	
	Unemployed	415 (10.38)		310 (11.39)	
	Other	596 (14.90)		433 (15.91)	
	Missing	16 (0.40)		4 (0.15)	
**Sexual orientation**					
	Straight	3242 (81.05)		2225 (81.74)	
	Other	654 (16.35)		441 (16.20)	
	Missing	104 (2.60)		56 (2.06)	
**Health insurance status**					
	No	729 (18.23)		457 (16.79)	
	Yes	3248 (81.20)		2259 (82.99)	
	Missing	23 (0.58)		6 (0.22)	
**Parent to ≥1 child**					
	No	2153 (53.83)		1596 (58.63)	
	Yes	1832 (45.80)		1123 (41.26)	
	Missing	15 (0.38)		3 (0.11)	
**US geographic region**					
	Midwest	811 (20.28)		583 (21.42)	
	Northeast	680 (17.00)		435 (15.98)	
	South	1576 (39.40)		1072 (39.38)	
	West	933 (23.33)		632 (23.22)	

^a^Percentages may not total 100% due to rounding.

## Discussion

We described the use and systematic application of four steps to examine the quality of responses and to clean data from a web-based survey completed by individuals who were part of a research panel, directly or through verified partners. There are several other strategies and techniques to screen and clean data (eg, missing data, stability of response patterns, outliers, and maximum long strings, among others [24,32,33,41]). The types and number of strategies to use for data quality assessment and data cleaning may vary depending on the content, length, and complexity of the web-based survey (eg, access to IP addresses, attention checks, speeder flags, and naivety of respondents, among others [24,25,41,42]). For example, we used straight-lining to assess consecutive identical responses, and we selected scales that contained reverse-coded items to conduct this step. Alternatively, investigators who do not have scales containing reverse-coded items might consider other methods to assess consecutive responses, such as long-string analysis [33] or maximum long-string assessment [32]. Another note for other researchers to consider is that we did not assess respondents’ IP addresses because we did not collect those data. There are benefits to collecting and examining IP addresses, such as identifying whether respondents took the survey more than once, but there may also be risks to collecting IP addresses, such as data protection and identity issues. Thus, investigators should consider risks and benefits when collecting IP addresses in their web-based surveys [25,43].

Other researchers have faced similar challenges when having to screen and filter out records with low-quality data collected from web-based surveys. Recently, researchers have reported disposing as much as three-quarters of data [44] or even their entire sample because over 90% of it was contaminated with fraudulent responses [35]. We lost about one-third of the original sample based on the criteria we used to clean the data. We recognize that losing this amount of data would be detrimental to an experimental design, but our study was observational and the final sample was sufficient for conducting our primary and exploratory analyses.

We hope that our step-by-step process encourages other research teams to systematically evaluate the integrity of their web-based survey data and use approaches to appropriately manage their data. Certainly, with the increased use of web-based surveys, it is imperative to evaluate data integrity and promote reporting transparency. A recent systematic review (n=80 studies) found that only 5% of the reviewed, published, web-based survey studies reported implementing checks to identify fraudulent data [45]. It is important to note that many panel companies may take steps to initially help ensure that panelists are participating in good faith (eg, not using bots to complete surveys) by using human intelligence tasks [3,4]. Many companies with research panels collect sociodemographic information about potential panelists and can send targeted study recruitment invitations based on the information initially reported to the company. As suggested by Dennis and colleagues [23], multiple entities have a responsibility and role in ensuring the integrity of the data [21,22].

This paper adds to the literature an applied, systematic example of data screening and management procedures that allow investigators to assess the quality of responses and eliminate invalid, fraudulent, or low-quality records. With the growing body of literature describing the application of quality assessment techniques and data cleaning approaches, this paper contributes an empirical example that could serve as a resource for other investigators and help streamline their data cleaning procedures. We have created a checklist as a tool for future studies (Textbox 1).

Cleaning the data from our web-based survey completed by panelists was a multistep and time-consuming process. However, after having invested time and effort into these quality assessment and data cleaning steps, we are more confident about the integrity of the remaining data in our final analytic sample. The final sample for manuscripts resulting from this survey data may vary depending on scientific goals and data analysis decisions (ie, handling of missingness, among others).

There were several key lessons learned from this experience. First, screening and quality checks should be in place both before and after collecting data from web-based survey platforms and research panelists. In future web-based surveys, our team plans to include attention checks and additional items to assess conflicting answers with the hope of both decreasing and identifying the number of responses that are careless, fraudulent, or both. An example of an attention check is one published by Chandler and colleagues [41], in which participants were asked to select “satisfied” from a list of response options. This item, or other similar items, could help to flag inattentive respondents and those providing invalid data. Another key lesson learned was that web-based survey programming requires extensive attention to detail. We recommend that other investigators consider the steps outlined in our checklist (Textbox 1) for development and testing of their survey. Furthermore, we recommend that investigators consider the features and the constraints of the software package where their online survey will be programmed. For example, software formatting, features, and functions may limit the way in which items can be displayed and, therefore, how participants interact with these items. Thus, we recommend that other research teams both understand the functions and capabilities of the survey package to be used and conduct usability tests with a small number of respondents who can pilot-test the web-based survey prior to its launch. Ideally, the test takers should be from the target population and not be part of the research team to avoid familiarity with the survey. This will allow the researchers to identify any components of the survey display that might be unclear or confusing to participants and allow for an opportunity to reformat or change the survey items prior to the survey launch. Lastly, we learned that there are multiple ways to apply data quality checks, including removing records that follow a pattern or a series of flags, removing records with multiple flags in a sequential way, and using a single flag to remove records. There are trade-offs to each of these. For example, removing participants with any one of a number of possible flags is likely to decrease the number of careless and poor-quality responses, thereby increasing the data quality while also decreasing the sample size and, thus, power for further analyses. On the other hand, removing only participants who show evidence of poor quality by all flags decreases the chance of wrongly removing respondents who took the survey seriously but had one or more flag, such as speed reading, at the cost of leaving in respondents who may have poor quality data by some but not all criteria. Ultimately, our team decided that using multiple types of flags in a sequential order was an efficient way to identify and remove records with invalid data. Additionally, in keeping with good reporting practices, we recommend that investigators use the CHERRIES checklist [28] to ensure that information reported in their manuscripts follow recommended reporting guidelines, such as the following: descriptions of their study design, survey development and pretesting, recruitment process, survey administration details, response rates, prevention of multiple survey entries, and data analysis procedures relevant to electronic surveys.

Web-based survey data collection and the use of research panels will likely continue to be used by research teams in the future. Certainly, there are pros and cons to collecting web-based survey data and recruiting participants from research panels. Developing a rigorous plan throughout the study, from survey inception and survey development to survey administration and statistical analyses; using multiple strategies for data quality checks and cleaning; and devoting time and attention can be effective components of improving data cleaning and management practice and consequently increasing the integrity of web-based survey data.

**The Checklist for E-Survey Data Integrity.**

Steps to develop and pretest an electronic survey:

- Provide clear instructions to participants and survey programmers
- Test skip and branching logic (ie, rules to jump to other items)
- Display items in a simple and logical way
- Display scales as individual items rather than in a table format
- Reduce the number of open-ended questions
- Reduce the use of complex fill-in tables
- Pretest the electronic survey in its final format for ease of administration and understanding (ideally with target population)
- Pretest the electronic survey for completion time
- Other (ie, other ways to tailor this checklist depending on needs and availability of data): ___________________________

Steps to prevent fraudulent responses (pre–data collection):

- Add attention checks (ie, ways to identify inattentive respondents)
- Add CAPTCHA or reCAPTCHA tasks
- Add speeder checks (ie, ways to identify fast respondents)
- Add items that can be used to verify responses or assess contradictions
- Collect IP address, geolocation, device, and browser used to access the survey
- Enable settings available within the web-based survey application to prevent multiple submissions and detect bots, among other issues
- Choose a platform that adheres to data privacy and compliance
- Other (ie, other ways to tailor this checklist depending on needs and availability of data): ___________________________

Steps to assess data integrity (post–data collection):

- Assess participant survey duration
- Check ranges of variables and examine responses that are clearly implausible
- Identify consecutive identical responses
- Identify contradictory responses
- Examine quality of open-ended responses
- Check IP address, geolocation, device, and browser information to identify multiple entries
- Other (ie, other ways to tailor this checklist depending on needs and availability of data): ___________________________

## Appendix

Multimedia Appendix 1 Descriptive information for Step 1—survey duration.

Multimedia Appendix 2 Scales used to examine consecutive identical responses (Step 2).

Multimedia Appendix 3 SAS code for Step 2 (consecutive identical responses).

Multimedia Appendix 4 SAS code for Step 3 (conflicting responses).

## Abbreviations

ASA: American Statistical Association
CHERRIES: Checklist for Reporting Results of Internet E-Surveys
HPV: human papillomavirus
PI: principal investigator
SCTR: South Carolina Clinical and Translational Science
