# Supplementary material for: Strategies and Lessons Learned During Cleaning of Data From Research Panel Participants: Cross-sectional Web-Based Health Behavior Survey Study
Source: JMIR Form Res. 2022 Jun 23;6(6):e35797. doi: 10.2196/35797 (PMC9264135; doi:10.2196/35797)
Supplement: Multimedia Appendix 1 [file formative_v6i6e35797_app1.docx]

**Multimedia Appendix 1**

**Table S1.** Descriptive information for Step 1 – survey duration.

| **Descriptive information** | **Original sample (in minutes)** | **Clean sample (in minutes)** |
| --- | --- | --- |
| N | 4,000 | 2,722 |
| Mean | 30.0 | 34.0 |
| Median | 18.2 | 21.2 |
| Minimum | 3.8 | 10.0 |
| Maximum ^a^ | 4958.6 | 4958.6 |
| 1st Quantile | 12.3 | 15.4 |
| 3rd Quantile | 28.6 | 31.7 |

^a^ Survey respondents were not removed due to taking longer to complete the survey, thus, the maximum duration time is the same for both samples.
