# Supplementary material for: Strategies and Lessons Learned During Cleaning of Data From Research Panel Participants: Cross-sectional Web-Based Health Behavior Survey Study
Source: JMIR Form Res. 2022 Jun 23;6(6):e35797. doi: 10.2196/35797 (PMC9264135; doi:10.2196/35797)
Supplement: Multimedia Appendix 2 [file formative_v6i6e35797_app2.docx]

**Multimedia Appendix 2**

**Table S1.** Scales used to examine consecutive identical responses (Step 2).

| *Scale* | *No. items* | *Reverse coded items* |
| --- | --- | --- |
| *HPV Knowledge*  Assessed knowledge about HPV.  Modified from Perez et al. 2016; modified Waller et al., 2013 | 19 | 7 false  12 true |
| *HPV Vaccine Knowledge*  Assessed knowledge about HPV vaccine.  Modified from Perez et al. 2016; modified Waller et al., 2013; modified Kasting et al., 2018 | 11 | 4 false  7 true |
| *Attitudes about Vaccines*  Assessed attitudes about vaccines (general)  From Zimet et al., 2010 | 11 | 5 negatively worded  6 positively worded |
| *Attitudes towards Seeking Medical Care*  Assessed attitudes about aspects of medical care seeking.  Shortened from Fischer et al., 2013—modified responses | 11 | 6 negatively worded  5 positively worded |
