# Supplementary material for: Strategies and Lessons Learned During Cleaning of Data From Research Panel Participants: Cross-sectional Web-Based Health Behavior Survey Study
Source: JMIR Form Res. 2022 Jun 23;6(6):e35797. doi: 10.2196/35797 (PMC9264135; doi:10.2196/35797)
Supplement: Multimedia Appendix 3 [file formative_v6i6e35797_app3.docx]

**Multimedia Appendix 3**

SAS code for Step 2 (consecutive identical responses).

**data** straight_line;

set short_time;

by id;

flag=**2**;

if ATTVACC1 in ('Strongly agree') and ATTVACC2 in ('Strongly agree' ) and ATTVACC3 in ('Strongly agree') and ATTVACC4 in ('Strongly agree' ) and

ATTVACC5 in ('Strongly agree') and ATTVACC6 in ('Strongly agree' ) and ATTVACC7 in ('Strongly agree' ) and

ATTVACC8 in ('Strongly agree') and ATTVACC9 in ('Strongly agree' ) and ATTVACC10 in ('Strongly agree' ) and

ATTVACC11 in ('Strongly agree') then output;

if ATTVACC1 in ('Strongly disagree') and ATTVACC2 in ('Strongly disagree') and ATTVACC3 in ('Strongly disagree') and ATTVACC4 in ('Strongly disagree') and

ATTVACC5 in ('Strongly disagree') and ATTVACC6 in ('Strongly disagree') and ATTVACC7 in ('Strongly disagree') and

ATTVACC8 in ('Strongly disagree') and ATTVACC9 in ('Strongly disagree') and ATTVACC10 in ('Strongly disagree') and

ATTVACC11 in ('Strongly disagree') then output;

if HPVKNOWL1 in ("True") and HPVKNOWL2 in ("True") and HPVKNOWL3 in ("True") and HPVKNOWL4 in ("True") and HPVKNOWL5

in ("True") and HPVKNOWL6 in ("True") and HPVKNOWL7 in ("True") and HPVKNOWL8 in ("True") and HPVKNOWL9 in ("True") and HPVKNOWL10 in

("True") and HPVKNOWL11 in ("True") and HPVKNOWL12 in ("True") and HPVKNOWL13 in ("True") and

HPVKNOWL14 in ("True") and HPVKNOWL15 in ("True") and HPVKNOWL16 in ("True") and HPVKNOWL17 in ("True") and

HPVKNOWL18 in ("True") and HPVKNOWL19 in ("True") then output;

if HPVKNOWL1 in ("False") and HPVKNOWL2 in ("False") and HPVKNOWL3 in ("False") and HPVKNOWL4 in ("False") and HPVKNOWL5

in ("False") and HPVKNOWL6 in ("False") and HPVKNOWL7 in ("False") and HPVKNOWL8 in ("False") and HPVKNOWL9 in ("False") and HPVKNOWL10 in

("False") and HPVKNOWL11 in ("False") and HPVKNOWL12 in ("False") and HPVKNOWL13 in ("False") and

HPVKNOWL14 in ("False") and HPVKNOWL15 in ("False") and HPVKNOWL16 in ("False") and HPVKNOWL17 in ("False") and

HPVKNOWL18 in ("False") and HPVKNOWL19 in ("False") then output;

if HPVVACCKNOWL1 in ("True") and HPVVACCKNOWL2 in ("True") and HPVVACCKNOWL3 in ("True") and HPVVACCKNOWL4 in ("True") and HPVVACCKNOWL5

in ("True") and HPVVACCKNOWL6 in ("True") and HPVVACCKNOWL7 in ("True") and HPVVACCKNOWL8 in ("True") and HPVVACCKNOWL9 in ("True") and HPVVACCKNOWL10 in

("True") and HPVVACCKNOWL11 in ("True")

then output;

if HPVVACCKNOWL1 in ("False") and HPVVACCKNOWL2 in ("False") and HPVVACCKNOWL3 in ("False") and HPVVACCKNOWL4 in ("False") and HPVVACCKNOWL5

in ("False") and HPVVACCKNOWL6 in ("False") and HPVVACCKNOWL7 in ("False") and HPVVACCKNOWL8 in ("False") and HPVVACCKNOWL9 in ("False") and HPVVACCKNOWL10 in ("False") and HPVVACCKNOWL11 in ("False")

then output;

if ATTMEDCARE1 in ("Agree") and ATTMEDCARE2 in ("Agree") and ATTMEDCARE3 in ("Agree") and ATTMEDCARE4 in ("Agree") and ATTMEDCARE5

in ("Agree") and ATTMEDCARE6 in ("Agree") and ATTMEDCARE7 in ("Agree") and ATTMEDCARE8 in ("Agree")

then output;

if ATTMEDCARE1 in ("Disagree") and ATTMEDCARE2 in ("Disagree") and ATTMEDCARE3 in ("Disagree") and ATTMEDCARE4 in ("Disagree") and ATTMEDCARE5

in ("Disagree") and ATTMEDCARE6 in ("Disagree") and ATTMEDCARE7 in ("Disagree") and ATTMEDCARE8 in ("Disagree")

then output;

**run**;

**proc** **sort** data=straight_line out=straight_line_id nodupkey;

by id;

**run**;
