# Supplementary material for: Strategies and Lessons Learned During Cleaning of Data From Research Panel Participants: Cross-sectional Web-Based Health Behavior Survey Study
Source: JMIR Form Res. 2022 Jun 23;6(6):e35797. doi: 10.2196/35797 (PMC9264135; doi:10.2196/35797)
Supplement: Multimedia Appendix 4 [file formative_v6i6e35797_app4.docx]

**Multimedia Appendix 4**

SAS code for Step 3 (conflicting responses).

**data** parent;

set hpv2;

by id;

flag=**3**;

if parent='Yes' and SEXEXP1='No' and DEM6 in ('Divorced' 'Married' 'Widowed' 'Separated') and

DEM8_4_TEXT not in ('A Sexual' 'A-Sexual' 'Aromantic/Asexual' 'Asexual' 'Asexual Spectrum' 'asexual')

then output;

**run**;
